# Effect of tube cooling on the osteogenic activity of concentrated platelet-rich fibrin in human periodontal ligament cells

**DOI:** 10.2340/biid.v13.46669

**Published:** 2026-09-03

**Authors:** Rucha Shah, Mavinakote Gowda Triveni, Baron Tarun Kumar, Raison Thomas, Hiral Karwa

**Affiliations:** Department of Periodontics, Bapuji Dental College and Hospital, Davangere, India

**Keywords:** Concentrated platelet-rich fibrin, cooling device, growth factors

## Abstract

**Objective:**

To compare the effect of concentrated platelet-rich fibrin (C-PRF) immediately after collection to C-PRF kept in a cooling device for about 90 min on the proliferation, osteogenic differentiation, and mineralization in human periodontal ligament cells (hPDLCs).

**Methods:**

Ten milliliters of blood without anticoagulants was collected in two tubes from 12 systemically healthy male volunteers and subjected to centrifugation at 700 RCF (relative centrifugal force)-max for 8 min for the test groups. C-PRF was collected immediately from the tubes of the first group, whereas in the second group, the tube was maintained at 4°C prior to blood collection and centrifugation, and following centrifugation, it was returned to a cooling device at 4°C for 90 min. This was then co-cultured with hPDLCs for 21 days. Cell viability and alkaline phosphatase levels were assessed on days 1, 7, 14, and 21. At the end of 21 days, Alizarin Red staining was done to assess the formation of mineralized nodules. All outcome assessments were conducted in three groups: C-PRF, cooled C-PRF, and a control group comprising hPDLCs cultured in an osteogenic basal culture medium without C-PRF and without any intervention.

**Statistical analysis:**

Linear mixed-effects models were used to analyze values across groups and time points.

**Results:**

Both the C-PRF group and cooled C-PRF group demonstrated significantly higher cell proliferation and alkaline phosphatase levels than the control group at day 7 and day 14. However, there was no statistically significant difference between the C-PRF and cooled C-PRF groups when compared for cell proliferation and alkaline phosphatase production at days 1, 7, 14, and 21 (*p* > 0.05). At the end of 21 days, both groups demonstrated significantly higher mineralization than the control group (*p* < 0.05); however, there was no difference between the C-PRF and the cooled C-PRF groups.

**Conclusion:**

Usage of C-PRF after storage at 4°C up to 90 min does not significantly alter cell proliferation, alkaline phosphatase production, and mineralized nodule formation compared with conventional C-PRF in hPDLCs.

## Introduction

In the last two decades, platelet-rich fibrin (PRF) has been widely researched as a regenerative biologic surgical additive for various applications in oral and periodontal pathologies such as in intrabony defects, sinus lifts, guided tissue regeneration, and recession coverage [1–3]. One limitation of the early PRF protocols, such as L-PRF (leukocyte-PRF) and A-PRF (advanced-PRF), is that they are obtained in a gel form, which is not very conducive to injection, unlike its predecessor, platelet-rich plasma (PRP), which is obtained in a liquid form and could be easily injected [4]. This limited the application of PRF in conditions where platelet concentrates could be injected, such as in facial esthetic procedures or injecting into the knee or temporomandibular joint for various therapeutic purposes.

To overcome this, Miron and co-workers in 2017 demonstrated the first liquid second-generation platelet concentrate, injectable PRF (i-PRF) [5]. This entails centrifugation of fresh blood without anticoagulants in plain tubes at 700 rpm for 3–4 min. Further, in 2021, a refined protocol of liquid PRF named concentrated PRF (C-PRF) was proposed, in which fresh blood was centrifuged at 700 g for 8 min to obtain increased cellularity and biologic function [6, 7]. Liquid protocols of PRF, such as injectable PRF and concentrated PRF, are now being actively researched and improved to obtain higher cellularity and increased and sustained release of growth factors [6, 8]. One of the key limitations of liquid PRF, in general, is its limited chairside time of around 15 min, beyond which it clots and is no longer usable in a liquid or injectable form. Miron and co-workers, in their recent article in 2022, proposed two methods of extending the chairside time of liquid C-PRF. This included the use of chemically modified polyethylene terephthalate (PET) tubes or cooling the tubes at 4°C, which led to an increase in the working time to 90 min, almost 270% [9].

However, these studies focused only on the handling properties and did not assess whether this storage time could affect the biologic activity of C-PRF. Hence, the aim of the present study was to assess and compare the effects of C-PRF immediately after collection to C-PRF kept in a cooling device for about 90 min on cell proliferation, osteogenic differentiation, and mineralization of human periodontal ligament cells (hPDLCs).

## Materials and methods

This cell culture research was authorized by the institutional review board and ethical committee (Ref no. BDC/exam/747) and was designed in line with the guidelines specified in the Declaration of Helsinki. A total of 12 systemically healthy, non-smoking men between the ages of 18 and 25 were selected as volunteers for the collection of blood. Subjects on any drugs affecting platelets, such as antiplatelet or anticoagulant drugs, were excluded from the study. Participants were subjected to a complete blood hemogram, and only those with all the hematological parameters within normal ranges were included. Each group consisted of six individual donors (*n* = 6 per group). For every donor, assays were performed in technical triplicate (C-PRF, cooled C-PRF) at each time point (days 1, 7, 14, and 21) to ensure reproducibility and accuracy of the measurements. Written consent was obtained from the participants after an explanation of the study procedure.

### C-PRF preparation

A Duo Quattro centrifuge (Process for PRF, Nice, France) with a fixed rotor angulation of 40° was used. Ten milliliters of venous blood was collected in plain plastic tubes and centrifuged at 700 × g for 8 min using the device’s relative centrifugal force (RCF) mode. As a result, the blood separated into three layers: the RBCs at the bottom, a buffy coat in the center immediately above the RBC layer, and comparatively clear platelet-poor plasma at the top. One to two milliliters of buffy coat, just above the RBC layer, was collected as C-PRF after the platelet-poor plasma was discarded.

In the cooled C-PRF protocol, blood was collected in refrigerated PRF tubes (4°C) to produce cooled PRF. Ten milliliters of blood was centrifuged immediately after collection for 8 min at 700 × g using the device’s RCF mode in plastic uncoated tubes (Process for PRF, Nice, France). The tubes were placed in the Pompac (Process for PRF, Nice, France) cooling equipment for 90 min at 4°C immediately after centrifugation. The handling time from removal of the tube from refrigeration, through centrifugation, to its placement back in the cooling device was approximately 10 min.

### Isolation and cultivation of hPDLCs

Teeth removed for orthodontic treatment were used to collect dental periodontal ligament cells [10]. Systemically and periodontally healthy male donors in the age range of 15–25 years undergoing extraction of maxillary first premolars for the purpose of orthodontic therapy were included for this purpose. Donors having any endodontic or periodontal issues with the tooth or its adjacent teeth were excluded. Written consent was obtained from the donors of the teeth for collection of the cells after explaining the purpose and methods of the study. The anonymity of the donors was ensured.

Following thorough cleaning of the teeth with phosphate-buffered saline, containing betadine and antibiotics (streptomycin + penicillin), Periodontal ligament (PDL) was carefully extracted from the tooth in a sterile Petri dish while adhering to all aseptic procedures. A portion of the tissue was treated for 1 hour at 37°C with 3 mg per mL collagenase type I and 4 mg per mL dispase in order to isolate hPDLCs. After adding Dulbecco’s Modified Eagle Medium (DMEM) augmented with 10% fetal bovine serum (FBS) to neutralize the enzymes, the mixture was filtered through a 70 μm cell strainer. This was done as previously described [10].

The separated cells were combined with alpha modification of minimal essential medium and then transferred to a 12-well plate. In order to track stem cell development, the medium was replaced twice a week while all samples were grown for at least 15 days. The tissues that showed fibroblast-like cells adhered to the plastic surface were removed for additional processing.

Cultures were kept in a humidified atmosphere with 95% air and 5% CO_2_ at 37°C. Cell growth and proliferation were evaluated in the resulting culture. Two to three subcultures were prepared (first and second passage). All the tests were performed in triplicate. All assays, including cell viability, alkaline phosphatase (ALP) activity, and Alizarin Red staining, were performed in all three groups: (1) C-PRF, (2) cooled C-PRF, and (3) control. The control group consisted of hPDLCs cultured in an osteogenic basal culture medium without C-PRF and without any intervention.

### Cell proliferation assay

The osteogenic treatment, which included adding DMEM and 10% FBS to each well, was applied to the PDL cells after they had been seeded into 6-well plates at a level of 10^4^ cells/well and cultivated for 8 hours. Then, treated cells were cultivated for a further 21 days. After cell attachment, 100 µL of C-PRF in its original liquid form from both groups was added to separate wells and soaked. C-PRF was added once at the beginning of each experimental period and was not replenished during the respective incubation interval. The same donor’s C-PRF was added for the triplicates. The plate was pre-incubated in a humidified incubator (e.g., at 37°C, 5% CO_2_). The supernatant fluid was collected at each study interval without disturbing the basal layer of cells, and the total culture volume per well was maintained at 1 mL at all times and replenished as needed throughout the 21-day period. As per the manufacturer’s guidelines, it was conducted using the cell counting kit (CCK)-8 (Merck, New Delhi, India). A microplate reader set to 450 nm was used to measure the optical density on days 1, 7, 14, and 21.

### ALP activity assessment

An ultrasensitive biochemical test (HiMedia Laboratories, Mumbai, India) was used to measure the ALP activity after the culture’s supernatant was extracted on days 1, 7, 14, and 21 [10].

In addition to 20 µL of the collected supernatant from each test group, a total of 960 µL of assay buffer and 20 µL of substrate solutions were incubated as per the manufacturer’s instructions. After that, absorbance was measured using a microplate reader at 405 nm. The following formula was used to calculate the values:

Alkaline phosphatase activity (units/mL) = Mean OD of test compound/Mean OD of Negative Control × DF × TV **/** 18.5 × EV.

Here, OD - optical density,

DF - dilution factor,

TV - total volume of the assay (mL)

EV - the volume of sample used

18.5 - millimolar extinction coefficient of pNPP at 405 nm.

### Alizarin Red S staining

After 21 days, cells were fixed with 100% cold methanol for 10 min, washed twice with sterile distilled water, and stained with a 2% Alizarin Red S solution for 15 min to measure the amount of mineralized nodules. The stained cultures were rinsed with distilled water to remove excess dye. On the culture dish, mineralized nodules were identified as red spots. For quantitative analysis, the dye was eluted using 300 µL of 33% glacial acetic acid, and the solution was collected for spectrophotometric analysis. Absorbance was measured at 415 nm, which was considered proportional to the extent of mineralization. Later, stained plates were scanned, and image-based assessment of mineralized nodules was performed as a qualitative/confirmatory measure.

The triplicate measurements from each sample were averaged to obtain one value prior to statistical analysis. Individual wells were not considered as independent observations.

SPSS version 20.0 was used for statistical analysis once all the data were entered into a proforma. Intergroup comparisons at different time intervals were performed using one-way analysis of variance. Post hoc comparisons were carried out using Bonferroni or Tukey’s test, as appropriate. A *p*-value of < 0.05 was considered statistically significant.

### Statistical analysis

Collected data were presented as mean ± standard deviation (SD) calculated from six donors per group. Cell proliferation (CCK-8) and ALP activity across days 1, 7, 14, and 21 were analyzed using a linear mixed-effects model (LMM) with group (C-PRF, cooled C-PRF, control) and time as fixed effects and donor as a random intercept, to account for the repeated contributions of the same six donors across all experimental groups. Alizarin Red S staining on day 21 was analyzed using an LMM with group as the fixed effect and donor as the random intercept. Post-hoc pairwise comparisons between groups were Bonferroni-corrected. Statistical analyses were conducted using Python 3.12. and jamovi (version 2.7.6).

## Results

**Figure 1:**
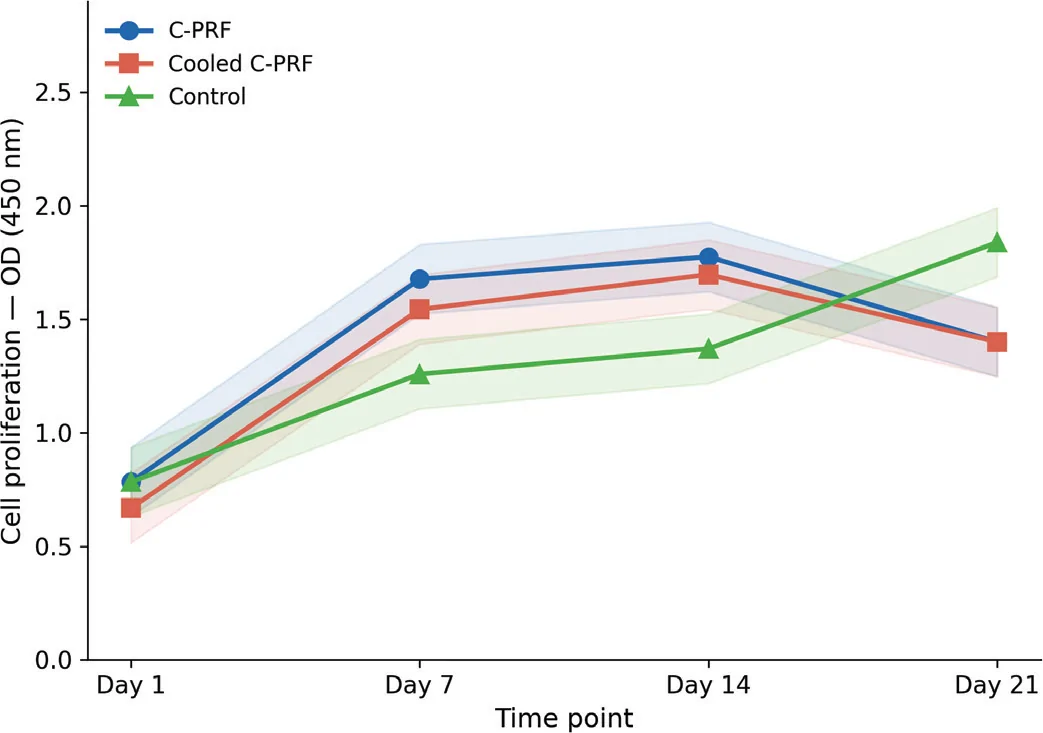
Estimated marginal means (± 95% CI) for CCK-8 optical density (450 nm) across days 1, 7, 14, and 21 for C-PRF (*n* = 6), cooled C-PRF (*n* = 6), and control (*n* = 6) groups. Shaded bands represent 95% confidence intervals derived from the linear mixed-effects model. C-PRF: concentrated platelet-rich fibrin; CCK: cell counting kit; OD: optical density.

CCK-8 optical density values are presented in Table 1. Cell proliferation was comparable between C-PRF and cooled C-PRF at all time points (*p* = 1.000 at each interval). Both PRF groups showed a peak response at day 14 (C-PRF: 1.78 ± 0.68; cooled C-PRF: 1.70 ± 0.15), followed by a decline at day 21, while the control group demonstrated a divergent pattern of continued increase through day 21 (1.84 ± 0.52). The overall Group × Time interaction was statistically significant (χ²(6) = 42.19, *p* < 0.001) for the divergence in the control trajectory. Pairwise comparisons between the PRF groups and control at individual time points did not reach significance after correction (*p* > 0.05 at all time points; Table 1; Figure 1).

**Table 1:** Cell proliferation (CCK-8 optical density at 450 nm) by group and time point: means, standard deviations, and pairwise *p*-values (*n* = 6 per group).

Group	Day 1	Day 7	Day 14	Day 21
	M (SD)	M (SD)	M (SD)	M (SD)
C-PRF	0.78 (0.25)	1.68 (0.51)	1.78 (0.68)	1.40 (0.23)
Cooled C-PRF	0.67 (0.08)	1.54 (0.21)	1.70 (0.15)	1.40 (0.13)
Control	0.78 (0.07)	1.26 (0.23)	1.37 (0.31)	1.84 (0.52)
Pairwise *p*	C-PRF vs. Cooled: 1.000 C-PRF vs. Control: 1.000 Cooled vs. Control: 0.080	C-PRF vs. Cooled: 1.000 C-PRF vs. Control: 0.291 Cooled vs. Control: 0.146	C-PRF vs. Cooled: 1.000 C-PRF vs. Control: 0.634 Cooled vs. Control: 0.128	C-PRF vs. Cooled: 1.000 C-PRF vs. Control: 0.257 Cooled vs. Control: 0.213

Note: Pairwise *p*-values are Bonferroni-corrected for three simultaneous comparisons per time point. M: mean; SD: standard deviation; C-PRF: concentrated platelet-rich fibrin.

**Figure 2:**
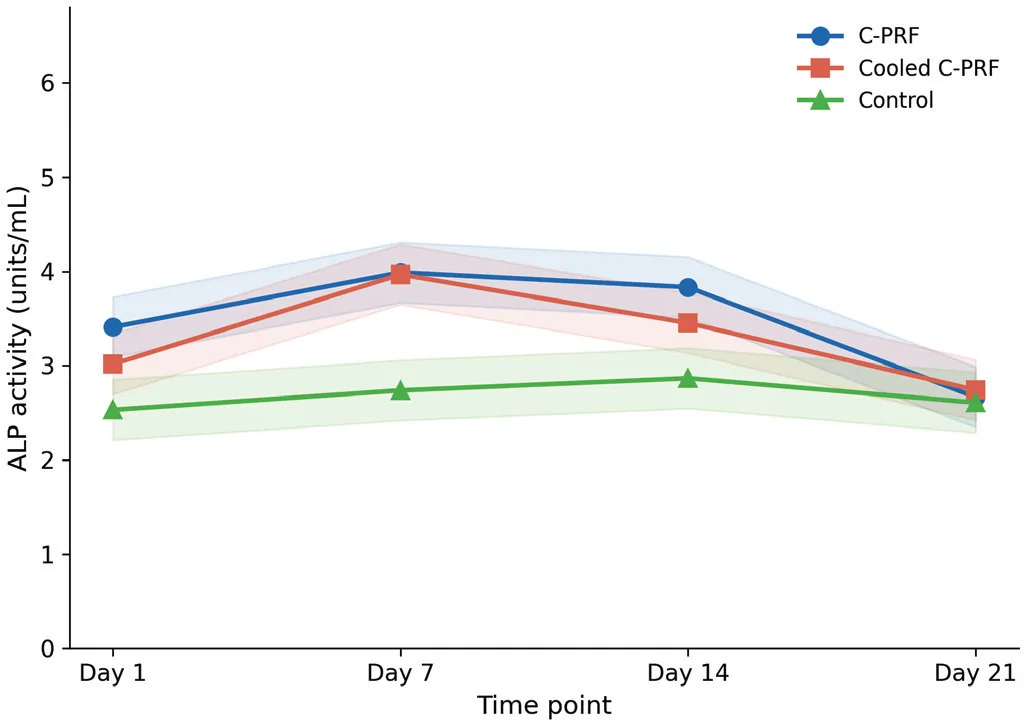
Estimated marginal means (± 95% CI) for alkaline phosphatase activity (units/mL) across days 1, 7, 14, and 21 for C-PRF (*n* = 6), cooled C-PRF (*n* = 6), and control (*n* = 6) groups. Shaded bands represent 95% confidence intervals derived from the linear mixed-effects model. C-PRF: concentrated platelet-rich fibrin; ALP: alkaline phosphatase.

ALP activity values are presented in Table 2. No significant differences in ALP activity were observed between the C-PRF and cooled C-PRF groups at any time point (*p* = 1.000 at each interval), indicating that the two preparations induced a comparable osteogenic differentiation response throughout the culture period. Both PRF groups exhibited peak activity at day 7 (C-PRF: 3.99 ± 1.19; cooled C-PRF: 3.97 ± 0.94), followed by a progressive decline through day 21. The control group maintained comparatively stable ALP activity across all time points (range: 2.53–2.87 units/mL; Table 2; Figure 2).

**Table 2:** Alkaline phosphatase activity (units/mL) by group and time point: means, standard deviations, and pairwise *p*-values (*n* = 6 per group).

Group	Day 1	Day 7	Day 14	Day 21
	M (SD)	M (SD)	M (SD)	M (SD)
C-PRF	3.41 (0.97)	3.99 (1.19)	3.83 (1.29)	2.67 (0.76)
Cooled C-PRF	3.02 (0.81)	3.97 (0.94)	3.45 (1.54)	2.74 (0.87)
Control	2.53 (0.80)	2.74 (0.67)	2.87 (0.71)	2.61 (1.03)
Pairwise *p*	C-PRF vs. Cooled: 1.000 C-PRF vs. Control: 0.356 Cooled vs. Control: 0.965	C-PRF vs. Cooled: 1.000 C-PRF vs. Control: 0.147 Cooled vs. Control: 0.078	C-PRF vs. Cooled: 1.000 C-PRF vs. Control: 0.420 Cooled vs. Control: 1.000	C-PRF vs. Cooled: 1.000 C-PRF vs. Control: 1.000 Cooled vs. Control: 1.000

Note: Pairwise *p*-values are Bonferroni-corrected for three simultaneous comparisons per time point. M: mean; SD: standard deviation; C-PRF: concentrated platelet-rich fibrin.

**Figure 3:**
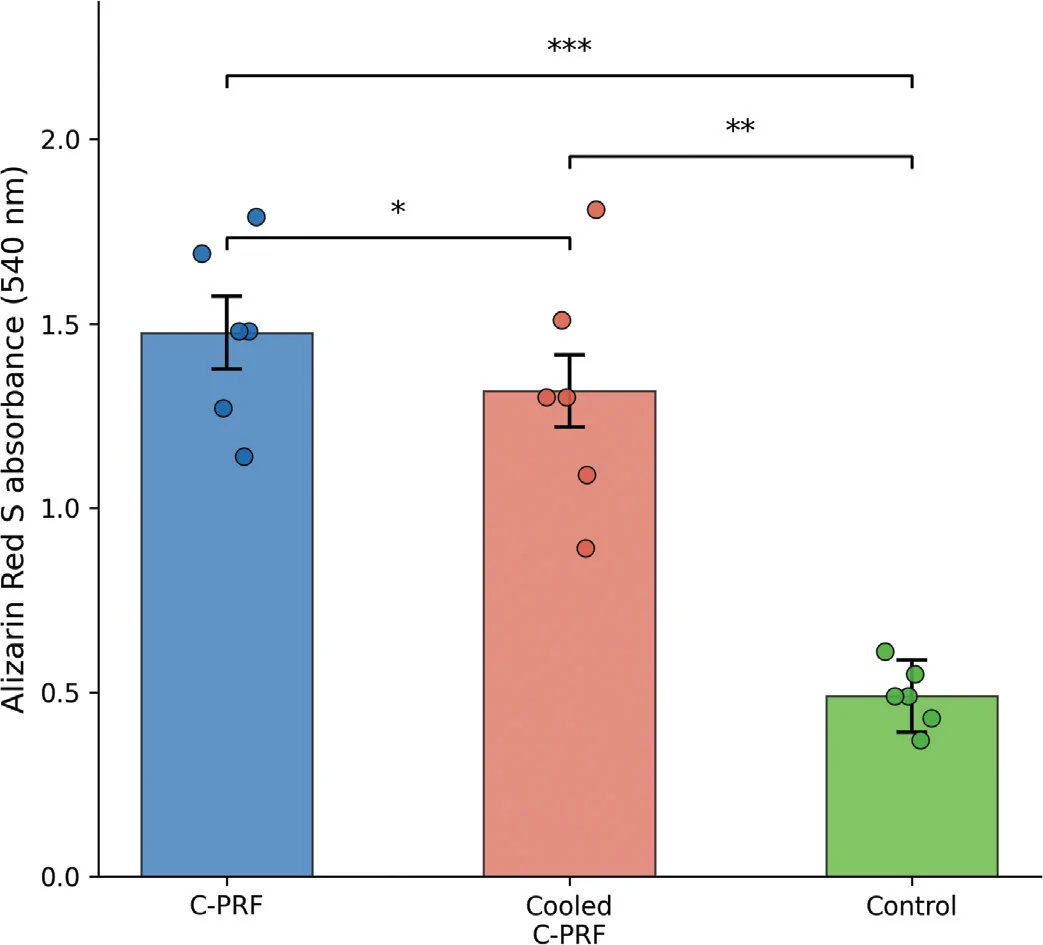
Alizarin Red S absorbance (540 nm) at day 21 for C-PRF (*n* = 6), cooled C-PRF (*n* = 6), and control (*n* = 6) groups. Bars represent group means; error bars denote 95% confidence intervals from the linear mixed-effects model. Individual donor values are superimposed. C-PRF: concentrated platelet-rich fibrin. Significance brackets indicate Bonferroni-corrected pairwise *p*-values: *p < 0.05; **p < 0.01; ***p < 0.001.

Alizarin Red staining on day 21 is presented in Table 3 and Figure 3. Both C-PRF (1.48 ± 0.25) and cooled C-PRF (1.32 ± 0.32) groups demonstrated significantly greater mineralized nodule formation than the control group (0.49 ± 0.09; both *p* ≤ 0.001). A statistically significant difference was observed between the C-PRF and cooled C-PRF groups (*p* = 0.025, Hedges’ *g* = 0.51); however, both protocols induced mineralization approximately threefold greater than the control, and the overall osteogenic response was well preserved in the cooled preparation.

**Table 3:** Alizarin Red S absorbance (540 nm) at day 21: group means, standard deviations, and pairwise comparisons (*n* = 6 per group).

Comparison	M (SD)	Mean difference	*p*	Hedges’ *g*
C-PRF	1.48 (0.25)	-	-	-
Cooled C-PRF	1.32 (0.32)	-	-	-
Control	0.49 (0.09)	-	-	-
*Pairwise comparisons*				
C-PRF vs. Cooled C-PRF	-	−0.16	0.025	0.51
C-PRF vs. Control	-	0.99	< 0.001	4.96
Cooled C-PRF vs. Control	-	0.83	0.001	3.25

Note: Mean difference = M (row group) – M (comparison group). Bonferroni correction applied for three simultaneous pairwise comparisons. Hedges’ *g* is reported as a measure of effect size. M: mean; SD: standard deviation; C-PRF: concentrated platelet-rich fibrin.

## Discussion

Over the last decade, the PRF protocol has evolved from gel-type L-PRF and A-PRF membranes to liquid PRF formulations (i-PRF, C-PRF) that aim to enhance the biologic activity of the concentrate by increasing the cellular content and the growth factor levels [4–7]. C-PRF, prepared at 700 g for 8 min, has been shown to yield higher concentrations of platelets, leukocytes, and macrophages, and increased growth factor release compared to the earlier PRF protocols [6, 7]. Liquid PRF is particularly attractive for injectable or hybrid graft applications, such as mixing with particulate bone or collagen scaffolds, or injecting around tissues such as soft tissues, around tooth roots, implants, and joints [8, 11]. However, a major limitation of these liquid formulations is their relatively short working time. Once blood without anticoagulant is drawn, the coagulation cascade proceeds and liquid PRF typically remains injectable for only about 10–15 min [6, 8]. This can be problematic for complex procedures, multiquadrant surgeries, or settings where multiple biomaterials are being prepared.

In order to determine if prolonging the working time of C-PRF by keeping the tubes in a cooling device has any impact on its osteogenic capacity in hPDLCs, the current *in vitro* investigation was conducted. Only male subjects were included to avoid any bias, as the sample size was limited. Recent studies by several groups of authors suggest significant differences in fibrin density and size of PRF membranes. In general, females produced almost 17% larger membranes than males [12]. When compared to freshly generated C-PRF, short-term storage of C-PRF at 4°C for up to 90 min did not considerably impair hPDLC proliferation, ALP activity, or mineralized nodule formation. The osteogenic capacity of C-PRF is maintained despite prolonged short-term storage (90 min) beyond the typical 10–15 min working time under cooling, as both groups demonstrated a progressive increase in ALP over 21 days and the formation of mineralized nodules by Alizarin Red staining. In the present study, ALP activity was measured in the supernatant without normalization, and as the values may be influenced by cell proliferation, they should be interpreted based on relative trends rather than absolute values.

Our results coincide with a number of recent studies that show PRF and its derivatives can stimulate the growth and osteogenic differentiation of cells obtained from the periodontal ligaments [6, 10, 13–15]. Different PRF preparations on hPDLCs and related cell types have been compared in a number of *in vitro* investigations. Red i-PRF and yellow i-PRF fractions had different effects according to Thanasrisuebwong and colleagues [13]. Red i-PRF promoted proliferation and migration, while yellow i-PRF stimulated early osteogenic differentiation and mineralization.

Farshidfar and co-workers demonstrated that i-PRF and its fractions can significantly upregulate osteogenic markers and mineralized nodule formation in periodontal ligament stem cells [14]. Li and co-workers reported that PRF exudate enhanced hPDLC proliferation, ALP activity, and calcium-nodular mineralization compared with untreated controls [10]. Similar results have been observed for other cell populations, where PRF increased ALP activity and expression of osteogenic markers, leading to enhanced mineral deposition. Recent literature on PRF mechanisms also demonstrated consistently increased *in vitro* activity of ALP and mineralization across several dental stem cell models [8, 15, 16].

In the present study, both fresh and cooled C-PRF groups demonstrated comparable optical density values for mineralization, confirming that C-PRF has a pro-osteogenic effect on the hPDLCs, in agreement with previous studies. The lack of significant difference between fresh and cooled C-PRF suggests that the cooling protocol did not significantly negate this biological benefit. This indicates that short-term storage at 4°C preserves the biologic activity of the PRF responsible for osteogenic stimulation, despite delaying coagulation. Cooling appears to act primarily as a handling aid – slowing fibrin polymerization and extending the injectable working window – without degrading platelets, leukocytes, or any signaling molecules. Thus, clinicians can benefit from improved chairside operating time while retaining the biologic activity of C-PRF.

Miron and co-workers recently addressed this by demonstrating that cooling of liquid PRF tubes using a dedicated device (Bio-Cool) or chemically modified PET tubes significantly extended the working time [9]. Their *in vitro* work showed that cooling increased liquid PRF working time by approximately 90 min (~270% improvement), without grossly affecting fibrin architecture. However, that study focused primarily on handling properties and fibrin structure; the downstream effects of cooling on cellular responses and osteogenic potential were not evaluated. These data indicate that cooling, as proposed by Miron and co-workers to extend the working time of PRF, does not appear to compromise the osteogenic signaling capacity of C-PRF in this cell model.

From a clinical point of view, the fact that C-PRF can be prepared and still be used after 90 min without the loss of biologic activity has several advantages. These include greater flexibility in performing long surgeries, where multiple injections or graft mixtures are prepared and applied sequentially. Our findings support the notion that cooling devices (such as those proposed by Miron and co-workers) can be adopted to extend working time, with no fear of any significant loss of osteogenic potential in hPDLCs.

Only healthy male donors were recruited for blood collection to minimize biological variability in PRF composition associated with sex-related differences in hematological parameters. Previous studies have demonstrated that gender can influence the characteristics and dimensions of PRF membranes, potentially affecting the cellular and growth-factor content of PRF preparations. Therefore, restricting donor recruitment to males allowed for greater standardization of the PRF samples used in this study. Nevertheless, this may limit the generalizability of the findings, and future studies should include both sexes to evaluate potential sex-related differences in PRF-mediated wound healing.

This study was designed as a pilot *in vitro* study using a single cell type (hPDLCs) in 2D culture. Future studies can be conducted in an *in vivo* environment or on containing polymicrobial biofilms. Additional markers studied previously, such as the effect on the physical properties, direct release of various growth factors or biomarkers, such as RUNX2, OCN, COL1, and OPN gene/protein expression, would provide a more detailed analysis of the effect of cooling on the overall properties [12, 17]. In-depth quantification of platelet, leukocyte, or growth factor concentrations before and after cooling would provide more direct evidence of preserved bioactivity. Other temperatures or longer storage, which may have a different impact on the biologic activity of PRF, may be investigated in the future.

## Conclusion

In summary, this study suggests that short-term collection in cooled tubes and storage of C-PRF at 4°C in a cooling device up to 90 min does not significantly diminish its ability to promote hPDLC proliferation, ALP activity, or mineralized nodule formation compared to freshly prepared C-PRF.

## Figures and Tables

**Table:** 

	Dr. Rucha Shah^1^	Dr. Triveni M G^2^	Dr. Baron Tarun Kumar^3^	Dr. Raison Thomas^4^	Dr. Hiral Karwa^5^
Concepts	✔	✔	✔	✔	✔
Design	✔	✔			
Definition of intellectual contents	✔	✔			
Literature search	✔	✔			
Clinical studies	✔	✔			
Experimental studies	✔	✔	✔	✔	
Data acquisition			✔	✔	✔
Data analysis			✔	✔	✔
Statistical analysis	✔		✔	✔	✔
Manuscript preparation			✔	✔	✔
Manuscript editing			✔	✔	✔
Manuscript review		✔	✔	✔	✔

## Data Availability

Additional data available on request from the corresponding author.
